# Gene model for the ortholog of *Sdr* in *Drosophila ananassae*

**DOI:** 10.17912/micropub.biology.001001

**Published:** 2025-08-16

**Authors:** Madeline L. Gruys, Anne E. Backlund, Logan Cohen, Veronica C. Gomes, Rida Mansoor, Jennifer A. Kennell, Jennifer Jemc, Nikolaos Tsotakos, Chinmay P. Rele, Laura K Reed

**Affiliations:** 1 University of Alabama, Tuscaloosa, AL USA; 2 Worcester State University, Worcester MA, USA; 3 Vassar College, Poughkeepsie, NY USA; 4 Loyola University Chicago, Chicago, IL USA; 5 Penn State Harrisburg, Middletown, PA USA

## Abstract

Gene model for the ortholog of *Secreted decoy of InR* ( *sdr* ) in the May 2011 (Agencourt dana_caf1/DanaCAF1) Genome Assembly (GenBank Accession: GCA_000005115.1 ) of *Drosophila ananassae* . This ortholog was characterized as part of a developing dataset to study the evolution of the Insulin/insulin-like growth factor signaling pathway (IIS) across the genus *Drosophila* using the Genomics Education Partnership gene annotation protocol for Course-based Undergraduate Research Experiences.

**Figure 1. Genomic neighborhood and gene model for Sdr in Drosophila ananassae:**
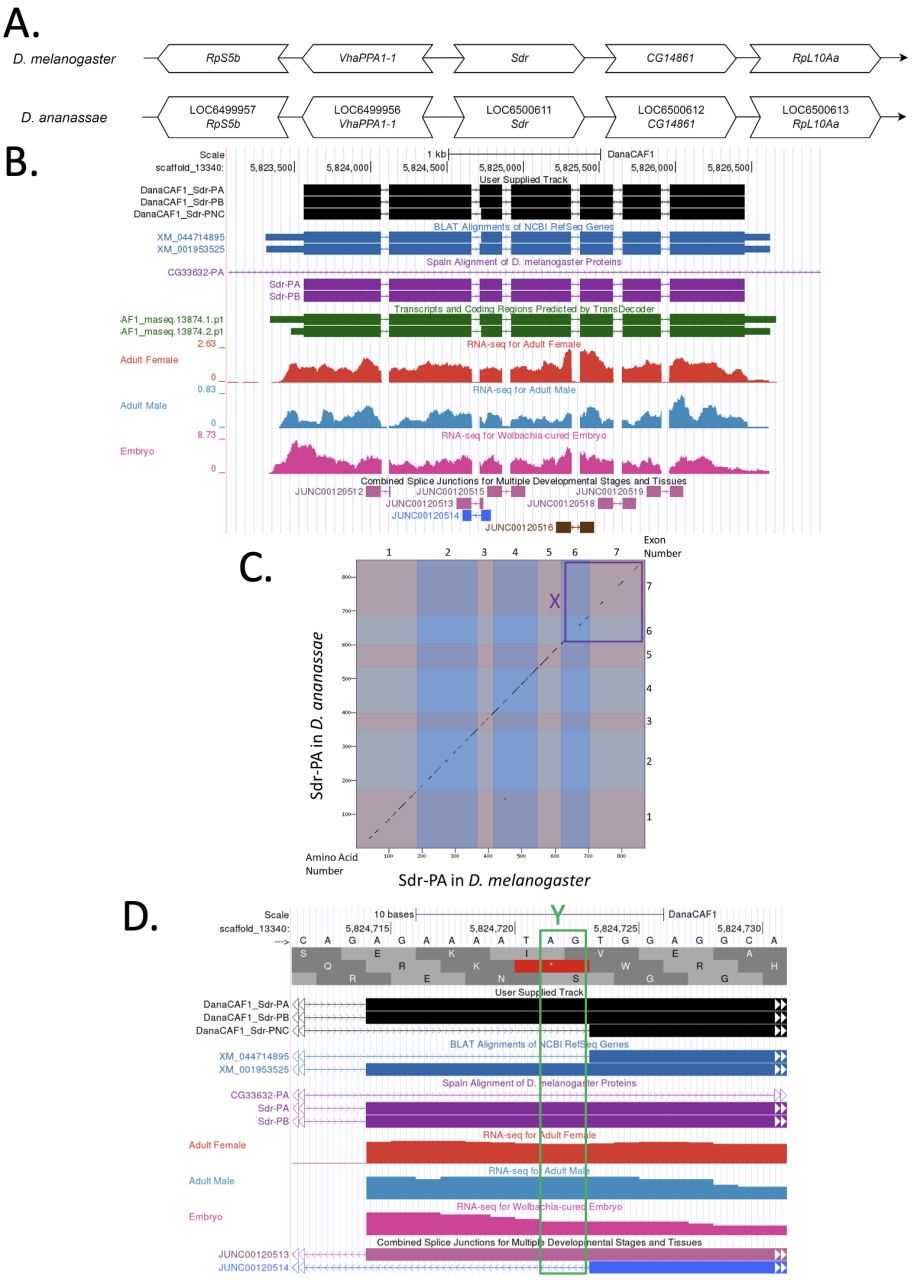
**
(A) Synteny comparison of the genomic neighborhoods for *Sdr* in *Drosophila melanogaster* and *D. ananassae* .** Thin underlying arrows indicate the DNA strand within which the gene– *Sdr* –is located in *D. melanogaster* (top) and *D. ananassae* (bottom). Thin arrows pointing to the right indicate that *Sdr* is on the positive (+) strand in *D. ananassae* and *D. melanogaster* . The wide gene arrows pointing in the same direction as *Sdr* are on the same strand relative to the thin underlying arrows, while wide gene arrows pointing in the opposite direction of *Sdr* are on the opposite strand relative to the thin underlying arrows. Wide gene arrows in *D. ananassae* indicate orthology to the corresponding gene in *D. melanogaster* . Gene symbols given in the *D. ananassae* gene arrows indicate the orthologous gene in *D. melanogaster* , while the locus identifiers are specific to *D. ananassae* . **(B) Gene Model in GEP UCSC Track Data Hub** (Raney et al., 2014) **.** The coding-regions of *Sdr* in *D. ananassae* are displayed in the User Supplied Track (black); coding CDSs are depicted by thick rectangles and introns by thin lines with arrows indicating the direction of transcription. Subsequent evidence tracks include BLAT Alignments of NCBI RefSeq Genes (dark blue, alignment of Ref-Seq genes for *D. ananassae* ), Spaln of D. melanogaster Proteins (purple, alignment of Ref-Seq proteins from *D. melanogaster* ), Transcripts and Coding Regions Predicted by TransDecoder (dark green), RNA-Seq from Adult Females, Adult Males and Wolbachia-cured Embryos (red, light blue and pink, respectively; alignment of Illumina RNA-Seq reads from *D. ananassae* ), and Splice Junctions Predicted by regtools using *D. ananassae* RNA-Seq ( SRP006203 , SRP007906 ; PRJNA257286 , PRJNA388952 ). Splice junctions shown have a read-depth of 13, 115-469 and 529 supporting reads in blue, pink and brown, respectively. Three splice junctions pertaining to the first and second introns (JUNC00120512, JUNC00120513 and JUNC00120514) appear to be on the opposite strand of the ortholog, however these splice junctions have been confirmed in the assembly ASM1763931v2/DanaRefSeq2. **(C) Dot Plot of Sdr-PA in *D. melanogaster* ( *x* -axis) vs. the orthologous peptide in *D. ananassae* ( *y* -axis).** Amino acid number is indicated along the left and bottom; CDS (coding exon) number is indicated along the top and right, and CDSs are also highlighted with alternating colors. Line breaks in the dot plot indicate mismatching amino acids at the specified location between species. A region of low conservation in the sixth and seventh CDS is highlighted by the purple box denoted X in the dot plot. **(D) Beginning of the third CDS of all isoforms of *Sdr* in the *D. ananassae* UCSC Genome Browser, displaying supporting evidence for the existence of a unique isoform, Sdr-PNC.** Evidence tracks are identical to those in the Gene Model (Figure 1B) with the addition of base position displayed at the top in black text and encoded amino acids in light and dark grey boxes below. The existence of the unique isoform denoted Sdr-PNC is supported by the splice junction (JUNC00120514) shown in blue and the presence of a canonical splice acceptor, highlighted by the green box denoted Y.

## Description

**Table:** 

*This article reports a predicted gene model generated by undergraduate work using a structured gene model annotation protocol defined by the Genomics Education Partnership (GEP; thegep.org) for Course-based Undergraduate Research Experience (CURE). The following information in this box may be repeated in other articles submitted by participants using the same GEP CURE protocol for annotating Drosophila species orthologs of Drosophila melanogaster genes in the insulin signaling pathway.* "In this GEP CURE protocol students use web-based tools to manually annotate genes in non-model *Drosophila* species based on orthology to genes in the well-annotated model organism fruitfly *Drosophila melanogaster* . The GEP uses web-based tools to allow undergraduates to participate in course-based research by generating manual annotations of genes in non-model species (Rele et al., 2023). Computational-based gene predictions in any organism are often improved by careful manual annotation and curation, allowing for more accurate analyses of gene and genome evolution (Mudge and Harrow 2016; Tello-Ruiz et al., 2019). These models of orthologous genes across species, such as the one presented here, then provide a reliable basis for further evolutionary genomic analyses when made available to the scientific community.” (Myers et al., 2024). “The particular gene ortholog described here was characterized as part of a developing dataset to study the evolution of the Insulin/insulin-like growth factor signaling pathway (IIS) across the genus *Drosophila* . The Insulin/insulin-like growth factor signaling pathway (IIS) is a highly conserved signaling pathway in animals and is central to mediating organismal responses to nutrients (Hietakangas and Cohen 2009; Grewal 2009).” (Myers et al., 2024). “ *D* . *ananassae* (NCBI:txid7217) is part of the *melanogaster* species group within the subgenus *Sophophora* of the genus *Drosophila* (Sturtevant 1939; Bock and Wheeler 1972). It was first described by Doeschall (1858). *D. ananassae* is circumtropical (Markow and O'Grady 2005; https://www.taxodros.uzh.ch, accessed 1 Feb 2023), and often associated with human settlement (Singh 2010). It has been extensively studied as a model for its cytogenetic and genetic characteristics, and in experimental evolution (Kikkawa 1938; Singh and Yadav 2015).” (Lawson et al., 2024).

We propose a gene model for the *D. ananassae* ortholog of the *D. melanogaster* *Secreted decoy of InR* ( *Sdr* ) gene. The genomic region of the ortholog corresponds to the uncharacterized protein XP_001953561.1 (Locus ID LOC6500611 ) in the May 2011 (Agencourt dana_caf1/DanaCAF1; ) Genome Assembly of *D. ananassae* ( GCA_000005115.1 ; Drosophila 12 Genomes Consortium et al., 2007). This model is based on RNA-Seq data from *D. ananassae* ( SRP006203 , SRP007906 ; PRJNA257286 , PRJNA388952 - Graveley et al., 2011) and *Sdr* in *D. melanogaster* using FlyBase release FB2022_04 ( GCA_000001215.4 ; Larkin et al., 2021; Gramates et al., 2022; Jenkins et al., 2022).

*Secreted decoy of InR* ( *Sdr* ) was first identified as a negative regulator of insulin signaling in *D. melanogaster* through a genetic screen (Okamoto et al., 2013). In *Drosophila* , insulin-like peptides ( *Ilps* ) are secreted ligands that bind to the insulin receptor ( *InR* ) to activate the insulin signaling pathway (Brogiolo et al., 2001; Ikeya et al., 2002). *Sdr* encodes a secreted protein that interacts with many of the Ilps, in particular Ilp3, but lacks the transmembrane and tyrosine kinase domains of the InR, thus acting as a secreted decoy (Okamoto et al., 2013). Mutants lacking *Sdr* develop into fertile adults that are larger due to increased insulin signaling and subsequent growth during larval stages (Okamoto et al., 2013; Millington et al., 2021). In the adult fly, *Sdr* is expressed in the surface glia covering the nervous system and is required for the maintenance of the blood-brain barrier and the blood-retina barrier (Kim et al., 2023).

***Synteny***

The reference gene, *Sdr* , occurs on chromosome 3R in *D. melanogaster* and is flanked upstream by *Ribosomal protein S5b* ( *RpS5b* ) and *Vacuolar H+ ATPase PPA1 subunit 1* ( *VhaPPA1-1* ) and downstream by *CG14861* and *Ribosomal protein L10Aa* ( *Rpl10Aa* ). The *tblastn* search of *D. melanogaster* Sdr-PA (query) against the *D. ananassae* (GenBank Accession: GCA_000005115.1 ) Genome Assembly (database) placed the putative ortholog of *Sdr* within scaffold scaffold_13340 ( CH902617.1 ) at locus LOC6500611 ( XP_001953561.1 )— with an E-value of 0.0 and a percent identity of 69.38%. Furthermore, the putative ortholog is flanked upstream by LOC6499957 ( XP_001953559.1 ) and LOC6499956 ( XP_001953560.1 ), which correspond to *RpS5b* and *VhaPPA1-1* in *D. melanogaster* (E-value: 7e-155 and 3e-146; identity: 90.87% and 97.64%, respectively, as determined by *blastp* ; Figure 1A, Altschul et al., 1990). The putative ortholog of *Sdr* is flanked downstream by LOC6500612 ( XP_001953563.1 ) and LOC6500613 ( XP_001953564.1 ), which correspond to *CG14861* and *Rpl10Aa* in *D. melanogaster* (E-value: 1e-82 and 3e-11; identity: 43.96% and 56.36%, respectively, as determined by *blastp* ). The putative ortholog assignment for *Sdr* in *D. ananassae* is supported by the following evidence: The genes surrounding the *Sdr* ortholog are orthologous to the genes at the same locus in *D. melanogaster* and local synteny is completely conserved, supported by results generated from *blastp* , so we conclude that LOC6500611 is the correct ortholog of *Sdr* in *D. ananassae* ( Figure 1A ).

***Protein Model***

*Sdr* in *D. ananassae* has three mRNA isoforms: *Sdr-RA* , *Sdr-RB* , and *Sdr-RNC* , which differs from *Sdr-RA* and *Sdr-RB* by the length of its third CDS ( Figure 1B ). mRNA isoforms ( *Sdr-RA, Sdr-RB* and *Sdr-RNC* ) contain seven CDSs each. Relative to the ortholog in *D. melanogaster* , the CDS number and protein isoform count are conserved, apart from the existence of the novel isoform Sdr-PNC in *D. ananassae* . The sequence of Sdr-PA in *D. ananassae* has 69.38% identity (E-value: 0.0) with the protein-coding isoform Sdr-PA in *D. melanogaster* , as determined by *blastp* ( Figure 1C ). Box X in purple encloses a region of low conservation in the sixth and seventh CDS of the ortholog displayed in the dot plot ( Figure 1C ). Coordinates of this curated gene model are stored by NCBI at GenBank accessions BK064543 , BK064544 , and BK064545 . These data are also archived in the CaltechDATA repository (see “Extended Data” section below).

***Special characteristics of the protein model***

**Novel isoform (Sdr-PNC) in *D. ananassae:***

*Sdr* contains one unique protein coding isoform encoded in *D. melanogaster* , by *Sdr-RA* and *Sdr-RB* , both of which are conserved in *D. ananassae* . A second unique isoform, denoted Sdr-PNC, appears to be present in the target species, differing only from *Sdr-RA* and *Sdr-RB* by the length of its third CDS. The existence of isoform Sdr-PNC is supported by the presence of a canonical splice acceptor (highlighted by the green box Y), Transcripts and Coding Regions Predicted by TransDecoder and the splice junction (JUNC00120514) shown in blue, with a read-depth of 13 ( Figure 1D ).

## Methods

Detailed methods including algorithms, database versions, and citations for the complete annotation process can be found in Rele et al. (2023). Briefly, students use the GEP instance of the UCSC Genome Browser v.435 (https://gander.wustl.edu; Kent WJ et al., 2002; Navarro Gonzalez et al., 2021) to examine the genomic neighborhood of their reference IIS gene in the *D. melanogaster* genome assembly (Aug. 2014; BDGP Release 6 + ISO1 MT/dm6). Students then retrieve the protein sequence for the *D. melanogaster* reference gene for a given isoform and run it using *tblastn* against their target *Drosophila* species genome assembly on the NCBI BLAST server (https://blast.ncbi.nlm.nih.gov/Blast.cgi; Altschul et al., 1990) to identify potential orthologs. To validate the potential ortholog, students compare the local genomic neighborhood of their potential ortholog with the genomic neighborhood of their reference gene in *D. melanogaster* . This local synteny analysis includes at minimum the two upstream and downstream genes relative to their putative ortholog. They also explore other sets of genomic evidence using multiple alignment tracks in the Genome Browser, including BLAT alignments of RefSeq Genes, Spaln alignment of *D. melanogaster* proteins, multiple gene prediction tracks (e.g., GeMoMa, Geneid, Augustus), and modENCODE RNA-Seq from the target species. Detailed explanation of how these lines of genomic evidenced are leveraged by students in gene model development are described in Rele et al. (2023). Genomic structure information (e.g., CDSs, intron-exon number and boundaries, number of isoforms) for the *D. melanogaster* reference gene is retrieved through the Gene Record Finder (https://gander.wustl.edu/~wilson/dmelgenerecord/index.html; Rele et al *.,* 2023). Approximate splice sites within the target gene are determined using *tblastn* using the CDSs from the *D. melanogaste* r reference gene. Coordinates of CDSs are then refined by examining aligned modENCODE RNA-Seq data, and by applying paradigms of molecular biology such as identifying canonical splice site sequences and ensuring the maintenance of an open reading frame across hypothesized splice sites. Students then confirm the biological validity of their target gene model using the Gene Model Checker (https://gander.wustl.edu/~wilson/genechecker/index.html; Rele et al., 2023), which compares the structure and translated sequence from their hypothesized target gene model against the *D. melanogaster* reference gene model. At least two independent models for a gene are generated by students under mentorship of their faculty course instructors. Those models are then reconciled by a third independent researcher mentored by the project leaders to produce the final model. Note: comparison of 5' and 3' UTR sequence information is not included in this GEP CURE protocol (Gruys et al., 2025).

## Data Availability

Description: A GFF, FASTA, and PEP of the model. Resource Type: Model. DOI: https://doi.org/10.22002/2e54s-eyd40
